# Evaluation of invasive breast cancer samples using a 12-chemokine gene expression score: correlation with clinical outcomes

**DOI:** 10.1186/s13058-017-0864-z

**Published:** 2017-06-19

**Authors:** Sangeetha Prabhakaran, Victoria T. Rizk, Zhenjun Ma, Chia-Ho Cheng, Anders E. Berglund, Dominico Coppola, Farah Khalil, James J. Mulé, Hatem H. Soliman

**Affiliations:** 1 H. Lee Moffitt Cancer Center and Research Institute, Tampa, FL USA; 2 University of South Florida Morsani College of Medicine, Tampa, FL USA

**Keywords:** Chemokines, Immunology, Gene expression score, Predictive marker, Breast cancer, Outcomes

## Abstract

**Background:**

A unique 12-chemokine gene expression score (CS) accurately predicted the presence of tumor-localized, ectopic lymph node-like structures (TL-ELNs) and improved overall survival (OS) in primary colorectal cancer and metastatic melanoma. We analyzed the correlation between CS, clinicopathological variables, molecular data, and 366 survival in Moffitt Cancer Center’s Total Cancer Care (TCC) patients with non-metastatic breast cancer.

**Methods:**

Affymetrix gene expression profiles were used to interrogate the CS by the principal component method. Breast tumors were classified as high or low score based on median split, and correlations between clinicopathologic variables, PAM50 molecular subtype, and ELN formation were analyzed using the TCC dataset. Differences in overall survival (OS) and recurrence-free survival (RFS) in the larger KM Plot breast cancer public datasets were compared using Kaplan-Meier curves.

**Results:**

We divided the Total Cancer Care (TCC) breast cancer patients into two groups of high or low CS. Mean CS was 0.24 (range, 2.2–2.1). Patients with higher CS were more likely to be white (172 vs. 159; *p* = 0.03), had poorly differentiated tumors (112 vs. 59; *p* <0.0001), ER/PR negative (41 vs. 26) and HER2 positive (36 vs. 19; *p* = 0.001), and contain TL-ELNs. Higher CS scores were also seen in the basal and HER2+ molecular subtypes. In the KM Plot breast cancer datasets higher CS patients demonstrated superior OS (HR = 0.73, *p* = 0.008) and RFS (HR 0.76, *p* = <0.0001), especially in basal and HER2+ patients.

**Conclusions:**

High CS breast tumors tend to be higher grade, basal or HER2+, and present more frequently in Caucasians. However, this group of patients also shows the presence of TL-ELNs within the tumor microenvironment and has better survival outcomes. The CS is a novel tool that can identify breast cancer patients with tumors of a unique intratumoral immune composition and better prognosis. Whether or not the CS is a predictive response marker in breast cancer patients undergoing immunotherapy remains to be determined.

**Electronic supplementary material:**

The online version of this article (doi:10.1186/s13058-017-0864-z) contains supplementary material, which is available to authorized users.

## Background

Breast cancer represents 14.0% of all new cancer cases in the United States, with 231,840 new cases and an estimated 40,290 deaths in 2015, comprising 6.8% of all cancer deaths [1]. Local and systemic treatments including surgery, chemotherapy, radiation, and endocrine therapy have all improved outcomes significantly for breast cancer patients [2]. However, the number of patients relapsing despite these treatments requires development of novel treatment modalities. One such modality that is garnering more attention recently is the use of immunotherapies [3]. Understanding how the various types of breast cancer interact with the immune system is important in informing us how to effectively utilize promising immune-oncology agents.

Although breast cancer is not perceived as a particularly immunogenic tumor when compared with melanoma and renal cell carcinoma as examples, molecular profiling of breast tumors has revealed that a subset demonstrate a high level of immunoregulatory gene activation [4]. Multiple investigators have reported that tumor-infiltrating lymphocytes and certain gene expression profiles related to immune signaling appear to have prognostic and/or predictive implications for breast cancer [especially the human epithelial growth factor receptor 2-positive (HER2+) type] [5–9]. These studies highlight the potential importance of the immune response in breast cancer patient outcomes. However, there are distinct types of immune cell infiltrates that can have different effects on tumor behavior. Characterization of the underlying mechanisms regulating immune infiltration in breast tumors can elucidate the key determinants for a successful host anti-tumor immune response. Secretion of chemokines within the tumor microenvironment and how certain co-morbidities like diabetes can affect the tumor chemokine milieu have gained attention as important factors that shape tumor lymphocyte infiltration [10, 11].

Chemokines act as trafficking signals for various immune cells and are important in orchestrating the spatial distribution of the immune response in a host. They also can directly affect the growth and progression of cancer cells [12]. Utilizing gene expression profiles can provide a more global assessment of immune signaling and cell populations using in silico methods such as CIBERSORT [13]. Certain chemokines have been associated with formation of a specific type of well-organized immune infiltrate known as tumor-localized, ectopic lymph node-like structures (TL-ELNs) [14]. It is hypothesized that these ELNs represent potent chemokine signaling gradients in the tumor microenvironment that attracts not only T cells but also activated B cells responding to specific tumor-associated antigens presented by co-localized dendritic cells [14].

Coppola and associates identified a unique 12-chemokine (CCL2, CCL3, CCL4, CCL5, CCL8, CCL18, CCL19, CCL21, CXCL9, CXCL10, CXCL11, and CXCL13) gene expression signature (GES) from a metagene grouping with overwhelming enrichment for immune-related and inflammation-related genes in primary colorectal cancer [15]. Messina and associates subsequently interrogated the 12-chemokine GES score (CS) across genomic arrays of 14,492 distinct solid tumors (primary and metastatic) of different histologies using the Total Cancer Care (TCC) database [16]. They found that this CS accurately predicted the presence of TL-ELNs and showed an association with improved overall survival in stage IV melanoma.

Since little was known about the effect of these aforementioned 12 chemokines on the breast tumor microenvironment, we sought to explore the relationship between the CS, presence of TL-ELNs, molecular subtype, and patient outcome in annotated breast cancer samples.

## Methods

### Patient inclusion criteria

A complete description of the TCC biobanking program has been previously published [17]. A retrospective review was performed on selected female patients with stage I to III breast cancer who were diagnosed between 1988 and 2012 and received primary surgery at the Moffitt Cancer Center. Patients may have received adjuvant therapies at Moffitt or at other locations. Snap-frozen tumor specimens from initially resected primary breast tumors were used for the gene expression profiles. Of the 813 unique gene expression files available, we included those from breast primary tumors only for which full clinical information was available. Patients chosen for study had available clinical and follow-up data within Moffitt Cancer Center’s electronic medical record system along with a genomic expression profile of their primary tumor. We excluded patients who had received any form of neoadjuvant therapy and with de novo metastatic disease, resulting in 366 patients in total.

### mRNA microarray analysis

The 12-chemokine score for these samples was extracted from the TCC database. The 12-chemokine score for TCC was calculated, in brief, as follows. Tumors from patients treated at the Moffitt Cancer Center were arrayed on modified Affymetrix HuRSTA-2a520709 GeneChips (Affymetrix, Santa Clara, CA, USA). Chips were normalized using iterative rank-order normalization (IRON) [18]. An RNA quality-related batch effect was identified in the resulting normalized data, which was removed by training a partial least squares (PLS) model [19] to the RNA integrity number (RIN) for each sample and then subtracting the first PLS component. The final 12-chemokine score across all tumors in TCC was calculated using the first component from a principal component analysis (PCA) model based on the 12-chemokine genes. The 13 following probe sets was used; CCL18: merck-NM_002988_at, CCL19: merck-NM_006274_at, CCL2: merck-NM_002982_at, CCL21: merck-NM_002989_at, CCL3: merck-D63785_x_at, CCL4: merck-NM_002984_at, CCL5: merck-NM_002985_at, CCL8: merck-NM_005623_at, CXCL10: merck-NM_001565_at, CXCL11: merck-NM_005409_at & merck2-NM_005409_at, CXCL13: merck-NM_006419_at, CXCL9: merck-NM_002416_at.

A two-sided Student *t* test and Bonferroni correction were used to test for differences in high (n = 183) and low (n = 183) gene expression in the 366 fully annotated specimens across the 12 genes in the chemokine gene expression signature. Principal component analysis was performed and visualized using Evince™. We also studied immune gene expression levels of B and T lymphocyte attenuator (BTLA), cluster of differentiation (CD)14, CD31, CD274 [programmed death ligand 1 or (PD-L1)], CD56, CD69, CTLA4, CXCL12, fibroblast activation protein (FAP), granzyme B, T cell immunoglobulin and mucin domain containing 3 (TIM3), indoleamine 2,3 dioxygenase (IDO1), interferon gamma (IFN-γ), interleukin (IL)2, IL10, IL4, IL6, Janus-associated kinase 1 (JAK1), lymphocyte activation gene 3 (LAG3), lymphocyte expansion molecule (LEM), homologous to lymphotoxin exhibits inducible expression and competes with HSV glycoprotein D for binding to herpesvirus entry mediator, a receptor expressed on T lymphocytes (LIGHT), MHC class I polypeptide-related sequence A (MICA), neural cell adhesion molecule 1 (NCAM1), nuclear factor kappa light chain enhancer of activated B cells (NFKB), nitrogen oxide synthase 1 (NOS1), programmed death 1 (PD1), perforin 1 (PRF1), signal transducer and activator of transcription 1 (STAT1), signal transducer and activator of transcription 3 (STAT3), and vascular endothelial growth factor A (VEGFA). We performed a two tailed Student’s *t* test with a Bonferroni correction for multiple testing, comparing mean expression levels of the above-noted immune genes between groups with high and low 12-chemokine gene expression scores.

### Pathologic analysis of tissue sections

Histological sections corresponding to 28 cases (prepared from the mirror image of the portion of tumor submitted for the mRNA microarray analysis) were retrieved from the Moffitt Cancer Center Anatomic Pathology Division’s repository as a pilot analysis to study correlation between the CS and tumor-localized, ectopic lymphoid node-like structures (TL-ELNs). Half of the specimens were from the top 10^th^ percentile 12-chemokine gene expression scores and half were from the bottom 10^th^ percentile. All of the specimens were 10% formalin fixed and paraffin embedded. Random representative hematoxylin and eosin (H&E)-stained sections through all selected tissue blocks were evaluated for the presence or absence of TL-ELNs. To further characterize the TL-ELNs, tissue sections were stained using the avidin-biotin complex method with retrieval under high pH. Pre-diluted monoclonal antibodies to CD3, CD4, CD8, and CD20 (Ventana Medical Systems, Tucson, AZ, USA) were used for the manual morphometric analysis of TL-ELNs by brightfield microscopy. To ensure pathologic concordance, two pathologists at our institution reviewed the tissue sections. Scores of 0 to 3 were assigned based on the following features: 0 = no lymphoid infiltrate noted in slide, 1 = 1 group lymphoid infiltrate, 2 = 2 groups of lymphoid infiltrate, and 3 = 3 or more groups of lymphoid infiltrate. Both pathologists were blinded as to the 12-chemokine gene expression scores of the individual samples. Clinical information was accessible only to the principal investigator and authorized collaborators, and all samples were anonymously coded before analysis. The Fisher’s exact test was used to test the association of 12-chemokine expression scores and H&E staining scores. The McNemar test was used to analyze the strength of agreement between the scoring methods of the two pathologists.

### Patient variables and outcomes analyses

We compared clinical and pathological factors of patients with low versus high 12-chemokine gene expression scores calculated by principal component analysis (determined by median split). Correlation between clinicopathologic factors and the 12-chemokine gene expression score was tested using the chi-square test with the exact method using Monte Carlo estimation. Kaplan-Meier curves were created for both overall survival (OS) and recurrence-free survival (RFS), and log-rank tests were used to compare 12-chemokine gene expression scores and 12-chemokine gene expression scores stratified by antibody status [estrogen receptor (ER) or progesterone receptor (PR) positive], ER and PR negative (with HER2 negative or missing), and HER2 positive. Multivariable survival models were fit using Cox proportional hazards model. Final models were chosen using backward selection, with a removal alpha of 0.05. All *p* values were two-sided unless otherwise stated and considered statistically significant at the 0.05 level. The final multivariate survival model incorporated age, pathologic stage, and ER status based on this criteria. All statistical analyses were performed using SAS (version 9.4; SAS Institute; Cary, NC, USA). A second log rank Kaplan Meier OS and RFS analysis was done on the larger KM Plot breast cancer dataset [20] due to the small number of events within the TCC cohort using the corresponding 12-chemokine gene probeset (216598_s_at (CCL2), 205114_s_at (MGC12815), 204103_at (CCL4), 204655_at (TCP228), 214038_at (CCL8), 210072_at (CCL19), 204606_at (CCL21), 203915_at (Humig), 204533_at (CXCL10), 211122_s_at (SCYB9B), 205242_at (CXCL13)) on the available Affymetrix arrays. In all KM Plot analyses the high versus low chemokine patients were split by mean upper tertile expression of the 12 chemokine genes computed across the entire dataset with equal weighting. Intrinsic subtypes in the KM Plot dataset are based on St. Gallen criteria using estrogen receptor 1 (ESR1), HER2, and antigen KI-67 (KI67). Prediction analysis of microarray 50 (PAM50) was used to classify breast cancer molecular subtypes within the TCC dataset (11). The estrogen, progesterone, and HER2 receptor status of the samples were classified by TCC pathologists using College of American Pathologists (CAP) criteria and obtained from the TCC database. Estrogen and progesterone receptors were positive if >1% immunohistochemical (IHC) staining was noted, and HER2 status was positive if 3+ by IHC or with a positive ratio by in situ hybridization assay. GraphPad Prism software (GraphPad Software, San Diego, CA, USA) was used to compare the mean chemokine gene expression scores with a Tukey test to obtain 95% confidence intervals between the molecular subtypes and receptor statuses by immunohistochemistry.

## Results

Our study included 366 TCC patients who met our inclusion criteria, with 183 patients in the low and 183 patients in the high 12-chemokine gene expression score groups. Low and high 12-chemokine gene expression score groups were compared regarding patient demographics, tumor characteristics, treatment variables, and survival status. The 12-chemokine gene expression scores ranged from -2.2 to 2.1 (median 0.24). The median age at diagnosis was 54.5 years (range, 24–90 years). The median follow-up was 66.3 months (range, 2.5–212.9 months). The chosen patient population was predominantly white (331 vs. 35), with 284 patients (77.6%) having ductal histology. There were higher numbers of moderately and poorly differentiated tumors, 148 patients (41.7%) and 171 patients (48.2%), respectively. Most of the chosen patients were ER or PR positive (212; 63.5%), followed by 67 patients (20.1%) who were negative for ER, PR, and HER2 or missing HER2 positivity. Fifty-five patients (16.5%) were HER2 positive. Patients mainly were diagnosed with stage II disease (176 patients; 51.8%). Of 366 patients, 314 (86.5%) received adjuvant systemic therapy after surgery, 71.9% were alive at time of data collection, and 83 patients (23.4%) had disease recurrence.

**Fig. 1:**
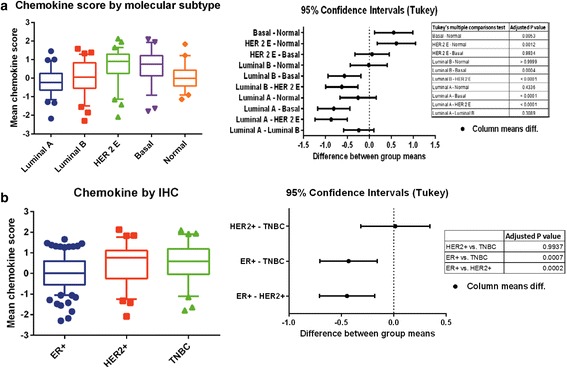
Comparison of chemokine score with (**a**) molecular subtype and (**b**) immunohistochemistry (*IHC*). *ER* estrogen receptor, *HER2* human epidermal growth factor receptor 2, *TNBC* triple-negative breast cancer

Patients in the high 12-chemokine gene expression group (versus low 12-chemokine gene expression group) were more likely to be Caucasian (172 vs. 159 patients; *p* = 0.0298), had higher rates of poorly differentiated/high grade tumors (112 vs. 59 patients; *p* < 0.0001), and were more likely to be ER/PR negative (41 vs. 26 patients) and HER2 positive (36 vs. 19 patients) (*p* = 0.001) (Table 1). When we compared 12-chemokine gene expression score with PAM50 molecular subtype, higher score correlated more with basal and HER2-positive subtypes (Fig. 1a). Based on receptor status by immunohistochemistry, higher 12-chemokine gene expression scores were associated with triple-negative breast cancer (*p* = 0.0007) and HER2-positive tumors (*p* = 0.0002) (Fig. 1b).

**Table 1:** Comparison of variables with 12-chemokine gene signature

		N (%)			
Variable	Level	Total	Low 12-chemokine	High 12-chemokine	*p* value
Race	White	331 (90.4%)	159 (86.9%)	172 (94%)	**0.03**
	Not white	35 (9.6%)	24 (13.1%)	11 (6%)	
Histology	Ductal	284 (77.6%)	136 (74.3%)	148 (80.9%)	0.371
	Lobular	52 (14.2%)	30 (16.4%)	22 (12%)	
	Others	30 (8.2%)	17 (9.3%)	13 (7.1%)	
Tumor grade	Well differentiated	36 (10.1%)	26 (14.9%)	10 (5.6%)	**<0.0001**
	Moderately differentiated	148 (41.7%)	90 (51.4%)	58 (32.2%)	
	Poorly/undifferentiated	171 (48.2%)	59 (33.7%)	112 (62.2%)	
Cancer status	Free (NED)	274 (82.5%)	138 (83.6%)	136 (81.4%)	0.668
	Not free of tumor	58 (17.5%)	27 (16.4%)	31 (18.6%)	
TNM stage	1	95 (27.9%)	49 (29.2%)	46 (26.7%)	0.709
	2	176 (51.8%)	83 (49.4%)	93 (54.1%)	
	3	69 (20.3%)	36 (21.4%)	33 (19.2%)	
Any adjuvant treatment	No	49 (13.5%)	23 (12.7%)	26 (14.3%)	0.755
	Yes	314 (86.5%)	158 (87.3%)	156 (85.7%)	
Receptor status	(ER+ or PR+)/(HER2- or HER2 missing)	212 (63.5%)	121 (72.9%)	91 (54.2%)	**0.001**
	ER-/PR-/(HER2- or missing)	67 (20.1%)	26 (15.7%)	41 (24.4%)	
	HER2+	55 (16.5%)	19 (11.4%)	36 (21.4%)	
Survival status	Alive	263 (71.9%)	133 (72.7%)	130 (71%)	0.82
	Dead	103 (28.1%)	50 (27.3%)	53 (29%)	
Recurrence status	No recurrence	272 (76.6%)	130 (73%)	142 (80.2%)	0.133
	Recurrence	83 (23.4%)	48 (27%)	35 (19.8%)	

*NED* no evidence of disease, *ER* estrogen receptor, *PR* progesterone receptor, *HER2* human epidermal growth factor receptor 2

A higher chemokine score was associated with Caucasian race, higher grade, ER- status, and HER2+ status

**Fig. 2:**
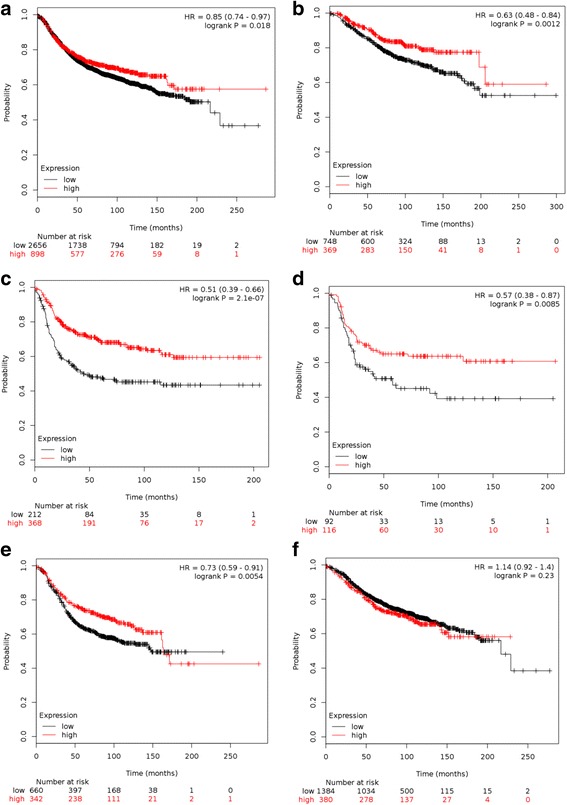
KM Plot Kaplan-Meier survival curves of (**a**) RFS in all patients (**b**) OS in all patients (**c**) RFS in basal subtype (**d**) HER-2 subtype (**e**) luminal B subtype (**f**) luminal A subtype patients. *Red line* = high chemokine group, *black line* = low chemokine group

The analysis of overall and recurrence-free survival in the KM Plot dataset demonstrated that patients with high 12-chemokine gene expression tumors had superior RFS (HR = 0.85, *p* = 0.018) and OS (HR = 0.63, *p* = <0.0012). The RFS was superior in patients with high 12-chemokine gene expression in the basal subtype (HR = 0.51, *p* = <0.0001), HER2 subtype (HR = 0.57, *p* = 0.0085), and luminal B subtype (HR 0.73, *p* = 0.0054). There was no RFS difference noted in the luminal A subtype (Fig. 2). A similar survival analysis was performed on the smaller TCC dataset, which showed a trend toward improved RFS in the HER2 subtype (Additional file 1). The number of OS and RFS events in the TCC dataset limited the power to fully evaluate the 12-chemokine gene expression score in relation to outcomes and was primarily used to evaluate associations between the 12-chemokine gene expression scores and clinicopathologic variables.

**Fig. 3:**
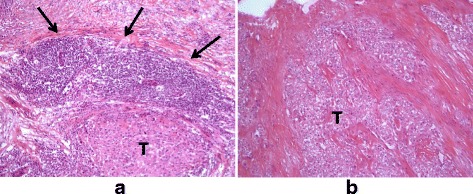
Representative examples of lymphoid aggregates. The highest 12-chemokine gene expression signature scored breast tumors (*T*) revealed peritumoral lymphocytic host response organized as one or more ectopic lymph node-like structures by H&E staining (*arrows*) (**a**). In contrast, the lowest 12-chemokine gene expression signature scored breast tumors were predominantly devoid of inflammatory infiltrate (**b**)

**Fig. 4:**
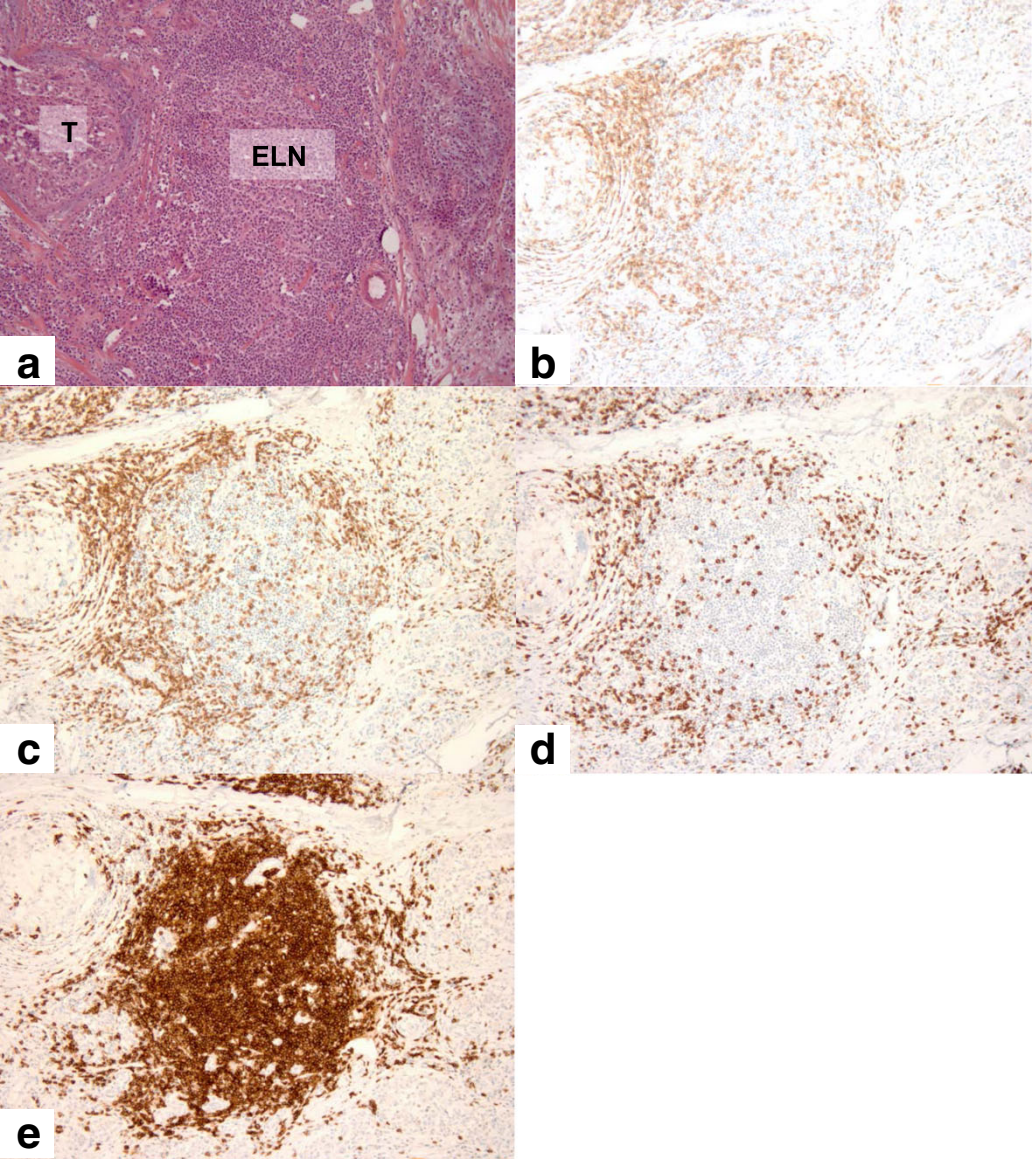
Analysis of primary breast tumors with H&E staining and IHC. Representative high-chemokine-scored breast slide revealed a marked peritumoral lymphocytic host response, organized as ectopic lymph node-like structures (*ELN*) by H&E staining (**a**) and by IHC (**b**–**e**). The immunohistochemical analysis of the lymphoid population highlighted the CD3+, CD4+, and CD8+ T cells (**b**, **c** and **d**) distributed in the parafollicular cortex or marginal zones and with some dispersion into the follicles while CD20 + B cells are concentrated in the center of the follicles (**e**). Magnification × 200

Of the 67 H&E tumor tissue slides analyzed histologically for immune cell infiltration, 28 were scored as 0 with absence of TL-ELNs and 39 were positive for TL-ELNs. Both the 12-chemokine gene expression score and immune cell staining score were associated with each other for both of the pathologists’ scores (*p* < 0.001). There were no significant differences between these two scoring methods (*p* = 0.052), and kappa strength of agreement of 0.6148 indicated substantial strength of agreement between the two pathologists (Tables 2 and 3). Immune cell infiltrate scores of 0 were noted in 31/34 (88.6%) and 28/28 (100%) of slides corresponding to low 12-chemokine gene expression score by each individual pathologist, respectively. Between 71 and 75% of tumors with high 12-chemokine gene expression scores were scored 1–3 for TL-ELNs on random sections evaluated for each tumor (Fig. 3). The immunohistochemistry stains of the TL-ELNs demonstrated perifollicular presence of CD3+ CD4+ and CD3+ CD8+ T cells with strong staining for CD20 centrally showing clustering of mature B cells (Fig. 4).

**Table 2:** Association of high and low chemokine scores and ELNs

			H&E stain score	
			0	1 + 2 + 3
H&E score	*p* value	Group level	N (%)	N (%)
1st pathologist	<0.001	High	4 (11.4)	24 (75)
		Low	31 (88.6)	8 (25)
2^nd^ pathologist	<0.001	High	0 (0.0)	28 (71.8)
		Low	28 (100)	11 (28.2)

Fisher’s exact test was used to test the association. The results indicate that the chemokine score and H&E score are associated with each other for both H&E score measurements

*ELNs* ectopic lymph nodes, *H&E* hematoxylin and eosin

**Table 3:** Scoring distribution between pathologists for ELNs

Table of two H&E stain scores			
1st pathologist	2nd pathologist		
Frequency percent row (%) col (%)	0	1 + 2 + 3	Total
0	25	3	28
	37.31	4.48	41.79
	89.29	10.71	
	71.43	9.38	
1 + 2 + 3	10	29	39
	14.93	43.28	58.21
	25.64	74.36	
	28.57	90.63	

McNemar’s test *p* = 0.0522  indicates no significant difference between these two scoring methods. Kappa strength of agreement was 0.6148 indicating substantial strength of agreement

*ELNs* ectopic lymph nodes, *H&E* hematoxylin and eosin

Gene expression levels of BTLA, CD274, CD69, CTLA-4, granzyme B, IDO, interferon gamma, IL10, IL2, IL6, LAG3, PD-1, PRF1, STAT1, LIGHT were all significantly higher in the 12-chemokine gene expression high group (Table 4). The enrichment of these genes indicates that the 12-chemokine gene expression score also identifies tumors with higher levels of an activated Th1-skewed cytotoxic T cell infiltrate.

**Table 4:** Comparison of immune gene expression between high versus low 12-gene chemokine signature scores in breast cancer

Immune genes	Mean values CS high	Mean values CS low	Adjusted *p* value
**BTLA**	**4.374103903**	**3.429457529**	**1.24E-30**
**BTLA**	**5.104717271**	**3.593084013**	**1.87E-41**
**CD14**	**10.54798175**	**10.07803072**	**1.00E-05**
**PD-L1**	**3.631025974**	**3.235645445**	**1.30E-12**
**PD-L1_2**	**7.857288587**	**6.090272006**	**1.47E-46**
**PD-L1_3**	**4.201128981**	**3.576133806**	**1.91E-19**
**CD69**	**8.500662981**	**7.112610181**	**9.03E-20**
**CTLA4**	**8.639718923**	**6.131656168**	**3.88E-59**
CXCL12	5.909474361	5.901681942	1
CXCL12_2	10.2584955	10.211514	1
CXCL12_3	10.37964834	10.27661866	1
FAP	10.84403166	10.76480457	1
**Granzyme B**	**8.749378335**	**5.759872516**	**6.87E-58**
**Granzyme B_2**	**8.799689613**	**5.545019987**	**3.45E-58**
**TIM3**	**8.841433445**	**8.110355729**	**1.97E-15**
**IDO1**	**8.966759748**	**5.712505632**	**6.00E-60**
**Interferon gamma**	**5.094852239**	**3.264361445**	**6.63E-38**
**IL10**	**3.717511361**	**3.298523542**	**6.72E-10**
**IL2**	**2.964185658**	**2.676063232**	**1.25E-10**
IL4	2.703027632	2.795378406	0.215457875
**IL6**	**6.746965877**	**5.733926619**	**7.80E-08**
JAK1	5.3000556	5.132389587	0.094531305
JAK1_2	9.330956665	9.094484413	0.024599705
JAK1_3	8.237782039	7.989419813	0.019509776
JAK1_4	10.80787464	10.7258152	1
**LAG3**	**6.455917471**	**4.991195632**	**1.00E-43**
LEM	3.0760666	3.059619619	1
LEM_2	6.110419432	6.080285335	1
LEM_3	9.176335942	9.183974716	1
LEM_4	9.772502419	9.798403587	1
MICA	8.81120191	8.799736368	1
MICA_2	4.690884181	4.882048935	1
MICA_3	4.891286084	5.075915484	1
MICA_4	8.791930277	8.781289103	1
MICA_5	4.693718703	4.827482226	1
MICA_6	9.571045	9.576738355	1
MICA_7	9.358850632	8.743868929	4.28E-07
CD56	5.580049794	5.914039671	1
CD56_2	4.629692439	4.864664335	1
CD56_3	3.710274097	3.817752942	0.453890338
CD56_4	5.035893445	5.352795768	1
CD56_5	5.70368229	6.044969677	1
NFKB	9.915169981	9.803810142	0.846175728
NOS1	2.902139606	2.921106129	1
NOS1_2	2.673781071	2.701705103	1
NOS1_3	2.993182194	2.955307058	1
**PD-1**	**4.767050374**	**4.533298226**	**6.38E-07**
CD31	8.753703548	8.316285368	4.15E-05
CD31_2	9.199703387	8.773391155	2.09E-06
CD31_3	11.64218184	11.36531043	0.001319025
CD31_4	9.182763284	8.7672612	1.42E-05
**PRF1**	**9.356943658**	**7.50412171**	**3.40E-47**
**STAT1**	**11.75385782**	**10.98503868**	**1.18E-40**
**STAT1_2**	**10.51426123**	**8.908289794**	**6.77E-47**
STAT1_3	4.153055619	4.103139297	1
STAT1_4	8.731805852	8.584214968	0.320434448
STAT1_5	10.17262105	10.11776818	1
**LIGHT**	**5.336076219**	**4.766262271**	**7.19E-20**
VEGA	8.954027852	8.900788865	1
VEGA_2	10.5322381	10.51156299	1
VEGA_3	9.817599665	9.782464335	1

Bolded gene probes are those with false discovery rate of <1% across all representative probes for a particular gene

## Discussion

The increasing awareness surrounding the importance of the host immune response in determining breast cancer outcomes provides new opportunities to integrate this information into treatment algorithms. Efforts to systematically describe the immune response in breast cancer by entities such as the TIL working group are critical to implementing this new system in the clinic [21]. However, given the complexity of the immune response and the need to personalize immunotherapy, it is becoming prudent to use molecular markers to dissect out what immune regulatory pathways are active in a given patient’s tumor [22]. The data presented herein indicate that certain chemokine genes can identify breast tumors enriched for tumor-localized, ectopic lymph node-like structures, and potentially provide a causal mechanism for why the tumor is inflamed in this manner.

Our study demonstrates that a 12-chemokine gene expression signature can identify a group of breast cancers with more favorable long-term outcomes. This is despite the fact that this group also contains greater number of tumors with traditionally adverse pathologic factors such as higher grade, ER negativity, and HER2 overexpression. In contrast to other immune infiltrate scoring methods, the chemokine score can provide a mechanistic explanation for why a particular tumor is forming TL-ELNs and exhibiting higher levels of activated T cell infiltrates. Another advantage of this approach is that chemokine scores can be obtained from limited core biopsies (i.e., prior to neoadjuvant therapy) while whole tissue sections would be required to histologically evaluate for the presence/absence of TL-ELNs in a tumor. An important question is what tumor-specific molecular features are conducive to the emergence of the high chemokine score phenotype. Future analyses should focus on analyzing other datasets combining RNA sequencing data that can provide information on mutational load and specific mutations or epitopes associated with a high chemokine gene expression score.

In our study, the TL-ELNs have the appearance of typical peripheral lymph nodes and are constructed of the necessary immune components, with CD3+, CD4+, and CD8+ T cells appearing in the parafollicular cortex or marginal zones and with some dispersion into the follicle and CD20+ B cells concentrated in the center of the follicle. The formation of these TL-ELNs is likely a different process compared to the lesser organized, dispersed infiltration of stromal tumor-infiltrating lymphocytes. Approximately 20% of invasive breast cancers contain perivascular TL-ELNs [20]. In particular, these infiltrates were associated with medullary breast cancers in one analysis, possibly accounting, in part, for its favorable prognosis [22].

Our study sheds light on the role of chemokine gene signaling in the tumor microenvironment and the formation of TL-ELNs, which potentially provides novel therapeutic opportunities. These may include manipulating TL-ELN-negative tumors to become TL-ELN-positive ones or isolating antibodies from the reactive B cell clones resident within TL-ELNs that potentially target tumor-associated antigens. Investigation of tumor chemokine gene expression scores in groups of breast cancer patients treated with checkpoint inhibitors and comparing its association with programmed death ligand 1 staining and clinical response is another possibility. In this respect, the chemokine score may prove useful to select patients for checkpoint blockade therapy. For chemokine-score-low breast tumors, increasing levels of key chemokines may ultimately prime those patients to respond more effectively to subsequent immunotherapies. Using immune gene expression signatures to personalize immunotherapy approaches could be critical in the future to maximizing clinical benefit in breast cancer patients.

## Conclusions

The 12-gene chemokine score evaluated in our study was associated with ectopic lymph node formation in breast tumors, increased gene expression of immune signaling pathways, and improved outcomes. The chemokine score should be further explored as a prognostic factor and predictive marker for emerging immunotherapy approaches in breast cancer patients.

## Additional file

Additional file 1: Outcomes (OS, RFS for entire cohort and RFS for HER2+) plus multivariate regression analysis of overall survival within the TCC dataset. (DOCX 119 kb)

## Abbreviations

BTLA: B and T lymphocyte attenuator
CD: Cluster of differentiation
CS: Chemokine score
CTLA4: Cytotoxic T lymphocyte associated protein 4
ELNs: Ectopic lymph nodes
ER: Estrogen receptor
ESR1: Estrogen receptor 1
FAP: Fibroblast-activation protein
GES: Gene expression signature
H&E: Hematoxylin and eosin
HER2: Human epithelial growth factor receptor 2
IDO1: Indoleamine 2,3 dioxygenase
IFN-γ: Interferon gamma
IHC: immunohistochemistry
IL: Interleukin
JAK1: Janus-associated kinase 1
KI67: Antigen KI-67
LAG3: Lymphocyte activation gene 3
LEM: Lymphocyte expansion molecule
LIGHT: Homologous to lymphotoxin exhibits inducible expression and competes with HSV glycoprotein D for binding to herpesvirus entry mediator, a receptor expressed on T lymphocytes
MICA: MHC class I polypeptide-related sequence A
NCAM1: Neural cell adhesion molecule 1
NFKB: Nuclear factor kappa light chain enhancer of activated B cells
NOS1: Nitrogen oxide synthase 1
OS: Overall survival
PAM50: Prediction analysis of microarray 50
PD1: Programmed death 1
PD-L1: Programmed death ligand 1
PR: Progesterone receptor
PRF1: Perforin 1
RFS: Recurrence-free survival
STAT1: Signal transducer and activator of transcription 1
STAT3: Signal transducer and activator of transcription 3
TCC: Total Cancer Care
TIM3: T cell immunoglobulin and mucin domain containing 3
VEGFA: Vascular endothelial growth factor A
