# Supplementary material for: Evaluation of invasive breast cancer samples using a 12-chemokine gene expression score: correlation with clinical outcomes
Source: Breast Cancer Res. 2017 Jun 19;19:71. doi: 10.1186/s13058-017-0864-z (PMC5477261; doi:10.1186/s13058-017-0864-z)
Supplement: Additional file 1: — Outcomes (OS, RFS for entire cohort and RFS for HER2+) plus multivariate regression analysis of overall survival within the TCC dataset. (DOCX 119 kb) [file 13058_2017_864_MOESM1_ESM.docx]

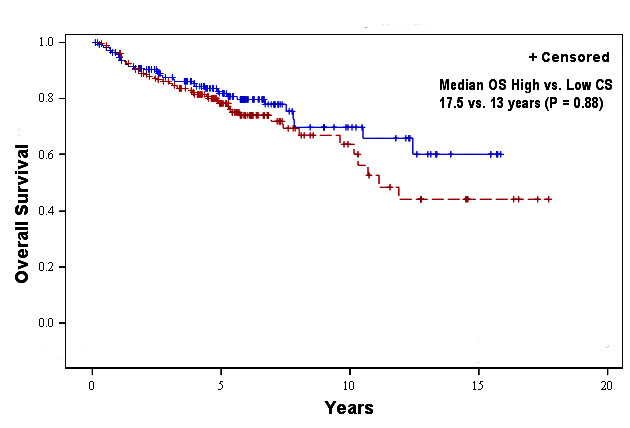

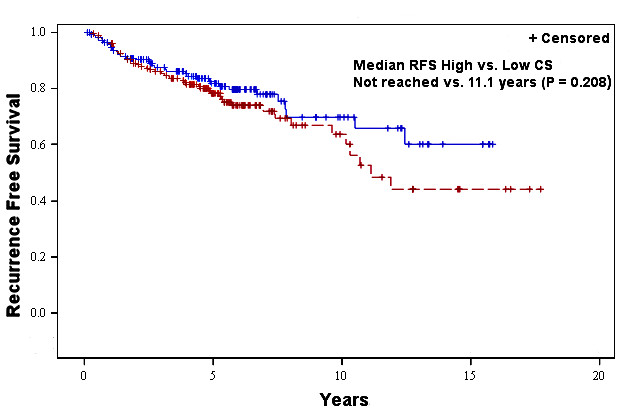


**(a)**

**(b)**


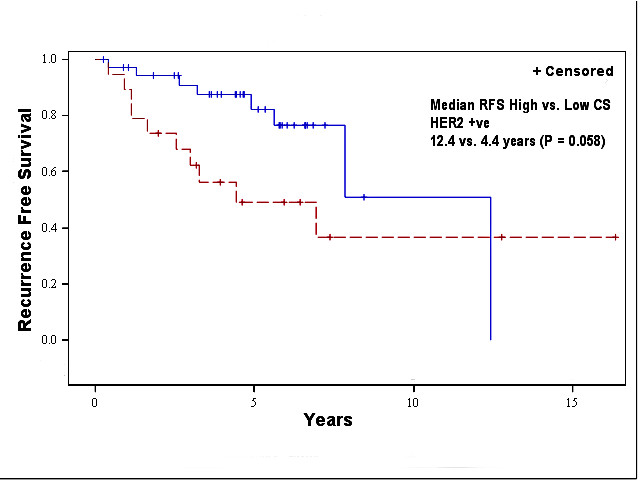


**(c)**

**Table 2: Univariate and significant multivariate analysis of Overall Survival**

| **Variable** | **Reference** | **Level** | **Univariate analysis** | | **Multivariate analysis** | |
| --- | --- | --- | --- | --- | --- | --- |
|  |  |  | **HR (95% CI)** | **P value** | **HR (95% CI)** | **P value** |
| 12 Chemokine |  |  | 0.96 (0.75,1.22) | 0.734 |  |  |
| 12 Chemokine (Median Split) | Low 12 Chemokine | High 12 Chemokine | 1.03 (0.7,1.52) | 0.88 |  |  |
| 12 Chemokine (10th and 90th percentile split) | Low 12 Chemokine | High 12 Chemokine | 0.7 (0.29,1.69) | 0.42 |  |  |
| Age at DX |  |  | 1.03 (1.02,1.05) | **<.0001** | 1.04 (1.02, 1.06) | **0.001** |
| Race | White | Not White | 1.63 (0.87,3.07) | 0.126 | 3.43 (1.55, 7.57) | **0.002** |
| Histology | Ductal | Lobular | 0.87 (0.5,1.52) | 0.622 |  |  |
|  | Ductal | Others | 0.55 (0.22,1.36) | 0.194 |  |  |
| Tumor Grade | Poorly/Undifferentiated | Well differentiated | 0.68 (0.32,1.42) | 0.3 |  |  |
|  | Poorly/Undifferentiated | Moderately differentiated | 0.6 (0.38,0.94) | **0.024** | 0.44 (0.24, 0.81) | **0.009** |
| Cancer Status | Free (NED) | Not free of tumor | 12.53 (7.86,19.98) | **<.0001** | 15.95 (8.98, 28.35) | **<.0001** |
| TNM Stage | 1 | 2 | 2.89 (1.42,5.87) | **<.0001** |  |  |
|  | 1 | 3 | 5.18 (2.45,10.95) |  |  |  |
| Any adjuvant treatment | No | Yes | 0.49 (0.3,0.8) | **0.004** | 0.43 (0.21, 0.88) | **0.02** |
| Receptor status | (ER+ or PR+)/(HER2- or HER2 Missing) | ER-/PR-/(HER2- or Missing) | 1.29 (0.76,2.21) | 0.349 |  |  |
|  | (ER+ or PR+)/(HER2- or HER2 Missing) | HER2+ | 1.72 (1.02,2.88) | **0.041** |  |  |

**Table 3: Univariate and significant multivariate analysis of Recurrence Free Survival between high and low chemokine groups**

| **Variable** | **Reference** | **Level** | **Univariate analysis** | | **Multivariate analysis** | |
| --- | --- | --- | --- | --- | --- | --- |
|  |  |  | **HR (95% CI)** | **P Value** | **HR (95% CI)** | **P Value** |
| 12 Chemokine |  |  | 0.89 (0.68,1.16) | 0.374 |  |  |
| Age at DX |  |  | 1.01 (0.99,1.03) | 0.199 |  |  |
| 12 Chemokine (Median Split) | Low 12 Chemokine | High 12 Chemokine | 0.76 (0.49,1.17) | 0.209 |  |  |
| 12 Chemokine (10th and 90th percentile Split) | Low 12 Chemokine | High 12 Chemokine | 0.72 (0.25,2.02) | 0.527 |  |  |
| Race | White | Not White | 1.57 (0.81,3.05) | 0.179 | 3.68 (1.67, 8.14) | **0.001** |
| Histology | Ductal | Lobular | 0.87 (0.47,1.61) | 0.656 |  |  |
|  | Ductal | Others | 0.26 (0.06,1.07) | 0.062 |  |  |
| Grade Differentiation | Poorly/Undifferentiated | Well differentiated | 0.49 (0.21,1.14) | 0.098 |  |  |
|  | Poorly/Undifferentiated | Moderately differentiated | 0.51 (0.31,0.83) | **0.007** |  |  |
| Cancer Status | Free (NED) | Not free of tumor | 15.92 (9.62,26.37) | **<.0001** | 25.08 (13.2, 47.64) | **<.0001** |
| TNM Stage | 1 | 2 | 2.11 (1.09,4.12) | **0.028** |  |  |
|  | 1 | 3 | 3.1 (1.48,6.48) | **0.003** |  |  |
| Any adjuvant treatment | No | Yes | 0.5 (0.28,0.89) | **0.019** | 0.31 (0.14, 0.65) | **0.002** |
| Receptor status | (ER+ or PR+)/(HER2- or HER2 Missing) | ER-/PR-/(HER2- or Missing) | 1.7 (0.98,2.94) | 0.061 | 2.99 (1.5, 5.95) | **0.002** |
|  | (ER+ or PR+)/(HER2- or HER2 Missing) | HER2+ | 2.11 (1.2,3.7) | **0.009** | 1.64 (0.83, 3.23) | 0.148 |
